# Gene expression profiling leads to discovery of correlation of matrix metalloproteinase 11 and heparanase 2 in breast cancer progression

**DOI:** 10.1186/s12885-015-1410-y

**Published:** 2015-06-18

**Authors:** Junjie Fu, Ravil Khaybullin, Yanping Zhang, Amy Xia, Xin Qi

**Affiliations:** 1 Department of Medicinal Chemistry, College of Pharmacy, University of Florida, 1600 SW Archer Rd, Health Science Center P5-31, Gainesville, FL 32610 USA; 2 Gene Expression and Genotyping, Interdisciplinary Center for Biotechnology Research, University of Florida, Gainesville, FL 32610 USA; 3 Columbia University, New York, NY 10027 USA

**Keywords:** Breast cancer, Gene expression profiling, Biomarker, MMP11, HPSE2

## Abstract

**Background:**

In order to identify biomarkers involved in breast cancer, gene expression profiling was conducted using human breast cancer tissues.

**Methods:**

Total RNAs were extracted from 150 clinical patient tissues covering three breast cancer subtypes (Luminal A, Luminal B, and Triple negative) as well as normal tissues. The expression profiles of a total of 50,739 genes were established from a training set of 32 samples using the Agilent Sure Print G3 Human Gene Expression Microarray technology. Data were analyzed using Agilent Gene Spring GX 12.6 software. The expression of several genes was validated using real-time RT-qPCR.

**Results:**

Data analysis with Agilent GeneSpring GX 12.6 software showed distinct expression patterns between cancer and normal tissue samples. A group of 28 promising genes were identified with ≥ 10-fold changes of expression level and *p*-values < 0.05. In particular, MMP11 and HPSE2 were closely examined due to the important roles they play in cancer cell growth and migration. Real-time RT-qPCR analyses of both training and testing sets validated the gene expression profiles of MMP11 and HPSE2.

**Conclusions:**

Our findings identified these 2 genes as a novel breast cancer biomarker gene set, which may facilitate the diagnosis and treatment in breast cancer clinical therapies.

## Background

Breast cancer is the second leading cause of death by cancer in women. It is estimated by the American Cancer Society that in 2014, approximately 232,670 new cases of invasive breast cancer will be diagnosed in women and up to 40,000 women will die from breast cancer in the United States alone [1].

There has been mounting evidence demonstrating that breast cancer is not one simple disease, but represents a heterogeneous group of tumors with different molecular subtypes, risk factors, clinical behaviors, and responses to treatments [2, 3]. Cancer biomarkers are increasingly being utilized for diagnostic, prognostic, and predictive purposes [4, 5]. Distinct molecular subtypes of breast cancer have been identified using the presence or absence of biomarkers, including estrogen receptors (ER+/ER-), progesterone receptors (PR+/PR-), and human epidermal growth factor 2 (HER2+/HER2-) [6–8]. The expression profiles of these three biomarkers are used to divide breast cancer into four subtypes: Luminal A, Luminal B, Triple negative (basal-like), and HER2 type [9, 10]. Among these subtypes, Luminal A is the most prevalent, accounting for 40 % of all breast cancers. Examples of biomarker-targeted therapy include when patients are given tamoxifen [11] for those with ER+ breast cancer and trastuzumab for those with HER2+ breast cancer, resulting in significantly improved prognosis [12]. However, these three classic molecular biomarkers are still insufficient, particularly considering that a significant portion of breast cancers falls under the triple negative breast cancer (TNBC) subtype [13, 14]. Therefore, claudin-low subtype and new biomarkers such as androgen receptors have been discovered for breast cancer [15, 16].

Gene signature, a group of genes whose combined expression pattern is uniquely characteristic of a biological phenotype or medical condition, can be a complement to classic prognostic factors to provide more accurate prognostic information [17]. During the past several decades, a number of gene signatures have been identified for breast cancer. For example, a 70-gene signature (MammaPrint; Agendia, Amsterdam, The Netherlands) and a 21-gene signature (OncoType; Genomic Health, Redwood City, CA) are being used in selected patients with early ER+ disease [18]. However, the 10-year results of ongoing clinical trials for testing the clinical benefit of gene signatures will not be fully available until 2020 [19]. Therefore, the identification of novel biomarkers and gene signatures in breast cancer remains highly essential, particularly considering that gene signatures in TNBC have not been fully developed yet.

Emerging technologies, such as gene expression profiling, are increasingly valued as powerful tools for new biomarker identification [20–24]. Data from gene expression profiling is generated from the analysis of hybridization microarray, which is a powerful method for high-throughput screening (HTS) of thousands of genes at one time. Previously, our group has studied the gene expression pattern of lung cancer using Affymetrix human exon array [25]. In this work, breast cancer gene expression profiling with mRNAs from clinical patient tissues was examined using the newly developed Agilent SurePrint G3 Human Gene Expression Microarray technology. This state-of-the-art high throughput platform takes advantage of the higher density available on the SurePrint G3 chip. Compared with other chips, the one we employed in this study exhibits a remarkably wide dynamic range (approximately 5 orders of magnitude), providing reliable detection of both low- and high-expressing genes. In addition, this technology requires low DNA input and the whole workflow is simple and straightforward. Genes of biological significance in breast cancers were identified via statistical analysis using GeneSpring 12.6 software. The expression levels of several selected genes were further confirmed using real-time reverse transcription quantitative polymerase chain reaction (RT-qPCR). Taken together, our gene expression profiling using the Agilent SurePrint G3 chip will contribute to the clinical diagnosis and treatment of breast cancer through the identification of novel breast cancer biomarkers.

## Methods

### Tissue samples

Tissue samples from clinical patients were acquired from the Clinical and Translational Science Institute (CTSI) Biorepository at University of Florida with all necessary ethical approval of collection and usage. All patients provided written informed consent for their tissue samples to be archived and used for research purposes. This study was approved by the University of Florida Institutional Review Board (IRB201200353) for breast cancer samples usage through UF CTSI Biorepository. A total of 150 tissue samples were included in this study, covering 3 subtypes of breast cancer (Luminal A, Lumina B, Triple negative) as well as normal tissue samples. All the human tissue samples were stored at −80 °C before RNA extraction.

### RNA preparation

Total RNA was isolated and purified from frozen tissue samples using Qiagen RNeasy Mini Kit, QIAshredder kit and RNase-Free DNase Set kit (Qiagen, Valencia, CA) following manufacturer's recommendations. The protocol includes: 1) homogenizing tissue by grinding in mortar with liquid nitrogen; 2) binding the homogenized tissue to the RNeasy Mini spin column; and 3) eliminating any trace amount of DNA using the DNase kit. The qualities of total RNA were strictly controlled by several parameters. The RNA extracts were first analyzed by Nanodrop 2000 (Thermo Fisher Scientific, Waltham, MA) and gel electrophoresis. RNA quality was determined by the ratios of A260/A280 (close to 2) and A260/A230 (close to 2), and the presence of 2 distinct ribosomal bands on gel electrophoresis. Qualified RNAs were further tested using Agilent 2100 Bioanalyzer (Agilent Technologies, Santa Clara, CA), and samples with 28S/18S RNA ratio > 1 were selected for gene expression profiling [26]. Thirty-two samples were finally tested, among which 2 samples C501 (Luminal A) and N513 (normal) were from the same patient, others are unmatched samples.

### Gene expression microarrays

Cyanine-3 (Cy3) labeled cRNA was prepared from 100 ng RNA using the One-Color Low Input Quick Amp labeling kit (Agilent, Valencia, CA) according to the manufacturer's instructions, and then purified by RNeasy Mini Kit (Qiagen, Valencia, CA) purification. Dye incorporation and cRNA yield were checked with the Nanodrop 2000 (Thermo Fisher Scientific, Waltham, MA). For hybridization, 0.6 μg of Cy3-labelled cRNA (specific activity > 8 pmol Cy3/μg cRNA) was fragmented at 60 °C for 30 min in a reaction volume of 25 μL containing 1X Agilent fragmentation buffer and 2X Agilent blocking agent following the manufacturers’ instructions. On completion of the fragmentation reaction, 25 μL of 2X Agilent hybridization buffer was added to the fragmentation mixture and hybridized to Agilent Whole Human Genome Oligo Microarrays (GPL17077) for 17 h at 65 °C in a rotating Agilent hybridization oven. After hybridization, microarrays were washed for 1 min at room temperature with GE Wash Buffer 1 (Agilent) and 1 min with 37 °C GE Wash buffer 2 (Agilent), then dried using Agilent stabilization and drying solution. Immediately after washing, slides were scanned on the Agilent DNA Microarray Scanner (G2505C) using1 color scan setting for 1x60k array slides (Scan Area 61 × 21 mm, Scan resolution 3 μM, Dye channel set to Green and Green PMT set to 100 %).

### Data normalization and quality control

This gene expression microarray data is deposited to the GEO repository and available via the accession number GSE57297 at http://www.ncbi.nlm.nih.gov/geo/query/acc.cgi?acc=GSE57297. The data were analyzed by GeneSpring 12.6 software (Agilent) and initial processing method was reported earlier [27]. The raw signals were log transformed and normalized using the Percentile shift normalization method, the value was set at 75th percentile. For each probe, the median of the log summarized values from all the samples was calculated and subtracted from each of the samples to get transformed baseline. The parameter values for experimental grouping were set as Luminal A, Luminal B, Triple negative, and Normal. Probes with intensity values below 20th percentile were filtered out using the “Filter Probesets by Expression” option.

### Differential expression analysis

Moderated *t*-test with Benjamin-Hochberg multiple testing corrections was used to calculate the *p*-value for the volcano plots. One-way ANOVA with asymptotic computation and Benjamin-Hochberg multiple testing corrections were used to calculate the *p*-value for the heat map. A *p*-value cutoff of 0.05 and a change of 2 fold or more were selected for gene analysis.

### Real-time RT-qPCR validation

cDNA was generated using SuperScript® VILO™ MasterMix (Invitrogen). All primers required were designed using Primer Premiere 6 software, and purchased from Integrated DNA Technologies (IDT). The real-time RT-qPCR reactions were prepared using SYBR® Select Master Mix (Life Technologies), and performed using BioRad CXF96 Real-Time PCR Detection System. The following conditions were used: 95 °C for 2 min, 40 cycles of 95 °C for 10 s and 60 °C for 1 min. Fold change of gene expression was calculated with the 2^-ΔΔC^_T_ method, using *β*-actin as the house keeping gene [28].

## Results

### Microarray gene expression profiling

**Fig. 1:**
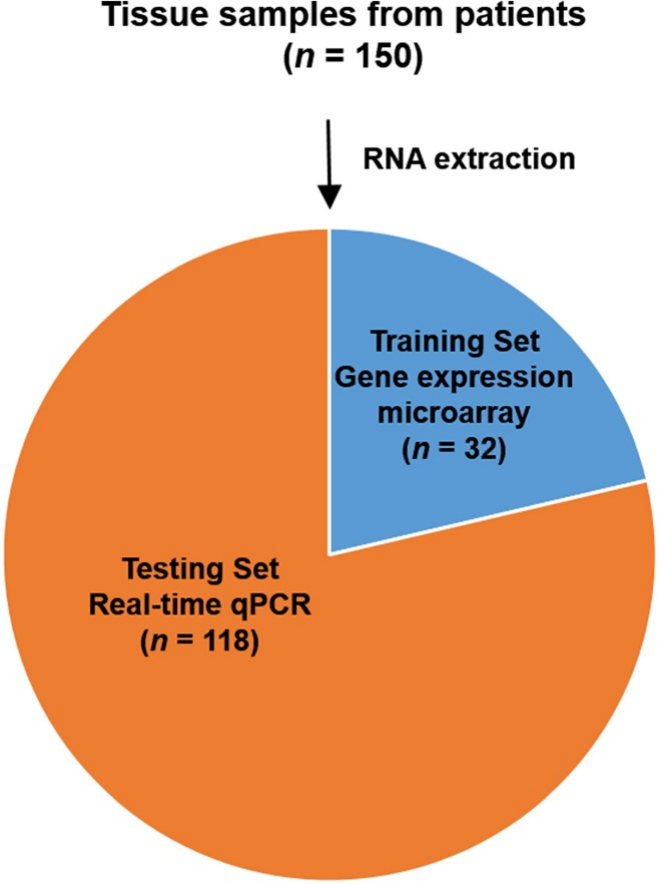
Schematic representation of the breast cancer gene expression profiling study. A total number of 150 tissue samples were examined. After RNA extraction and purification, 32 RNA samples were selected as the training set for microarray gene expression profiling. The remaining 118 samples were used as a testing set to validate the gene expression results by real-time RT-qPCR

The 150 tissue samples in the study included all three subtypes of breast cancer (Luminal A, Lumina B, Triple negative) as well as normal tissue samples. After RNA extraction and purification, 32 RNA samples (19 from Luminal A, 3 from Luminal B, 3 from Triple negative, and 7 normal samples) were selected as the training set for microarray gene expression profiling. The remaining 118 samples were used as a testing set to validate the gene expression results from the training set (Fig. 1). In the training set, 2 samples C501 (Luminal A) and N513 (normal) were from the same patient, others are unmatched samples.

As many as 50,739 probes were used to detect mRNA expression levels for each RNA sample using SurePrint G3 Human Gene Expression 8 × 60 K v2 Microarray Kit, and the data were analyzed using Gene Spring 12.6 (Agilent). Probes with intensity values below 20th percentile were filtered out, resulting in 38,432 genes, which were used for differential expression analysis. Out of the 38,432 genes, the expressions of 4569 genes were found to be statistically significantly different after One-way ANOVA test [29] with a corrected *p*-value less than 0.05. Furthermore, 1061 genes showed fold changes of expression (compared with normal control) larger than 2 in all three breast cancer subtypes (Luminal A, Luminal B, and Triple negative) [30]. Among these 1061 genes, most of them were consistently up-regulated (217 genes) or down-regulated (720 genes) in all three subtypes, while the other 124 genes showed different expression patterns among different breast cancer subtypes (Table 1). It is notable that in most cases, the gene regulation patterns were the same between Luminal A and Luminal B subtype samples, as only 9 genes displayed different regulation between these 2 subtypes. This is consistent with the report that Luminal A and Luminal B share a significant number of characteristics [9, 10]. For example, both Luminal A and Luminal B subtype are characterized by expression of ER, PR, and other genes associated with ER activation.

**Table 1:** The regulation pattern of the 1061 genes^a^ among three breast cancer subtypes

Number of genes	Gene regulation		
	Luminal A	Luminal B	Triple negative
217	up^b^	up	up
720	down^b^	down	down
60	up	up	down
55	down	down	up
2	down	up	up
7	down	up	down

^a^Genes with corrected *p* < 0.05 and fold changes ≥ 2 in all three breast cancer subtypes were selected

^b^The classification as “up” or “down” refers to fold changes with respect to normal tissues

**Fig. 2:**
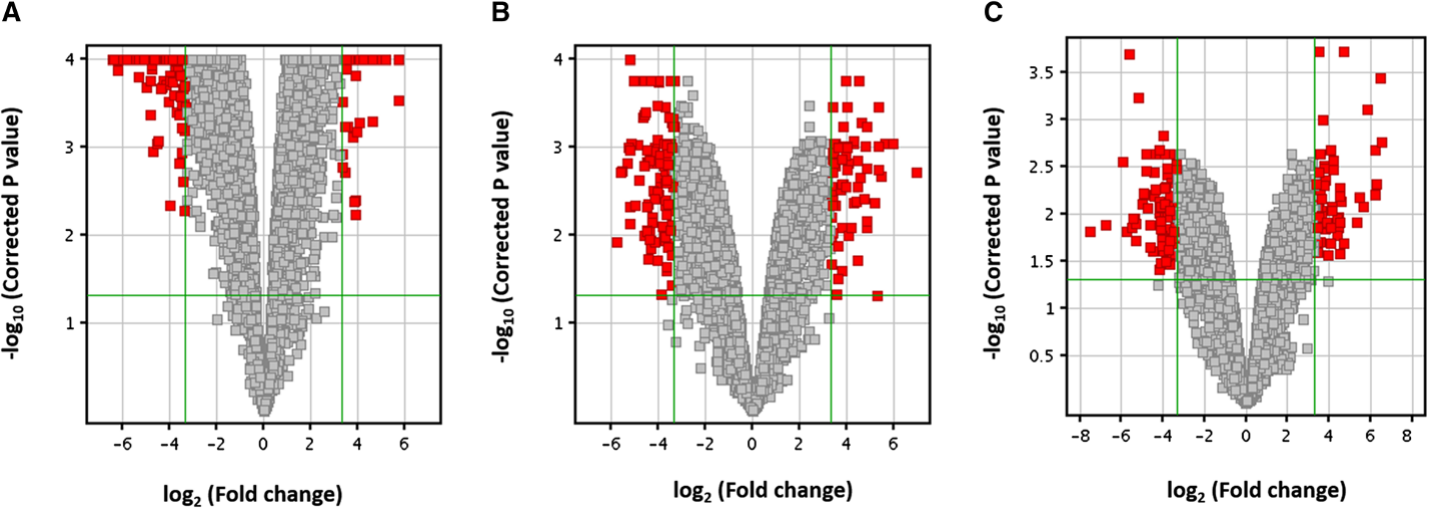
Volcano plots. The distribution of the gene expression fold changes and corrected *p*-values in each subgroup **a** Luminal A, **b** Luminal B, and **c** Triple negative compared with normal controls were shown. A total number of 4569 genes with *p*-value < 0.05 were used for the analysis. Genes with absolute fold change ≥ 10 and *p*-value < 0.05 are indicated in red. Plots are generated using Gene Spring 12.6 with moderated *t*-test and Benjamini-Hochberg testing correction

**Fig. 3:**
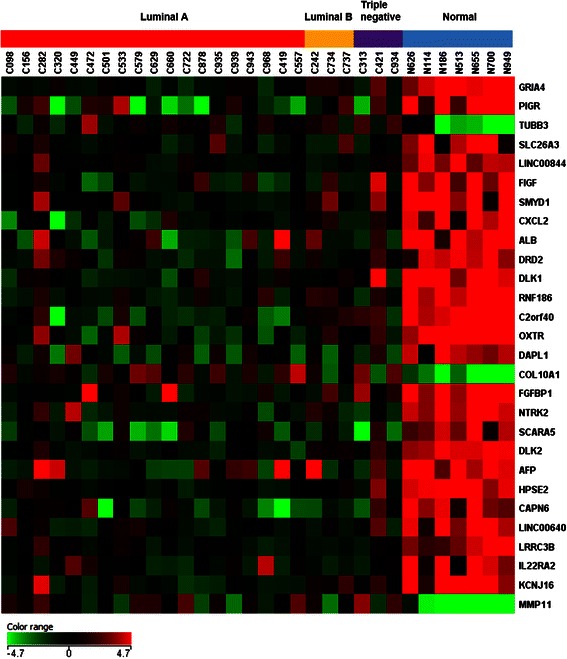
Heat map. The expression patterns of 28 genes out of 50,739 biological probes after one-way ANOVA test with a corrected *p*-value < 0.05 and fold change ≥ 10 in all three breast cancer subtypes were shown in the heat map using Gene Spring 12.6 software. The heat map indicates up-regulation (*red*), down-regulation (*green*), and mean gene expression (*black*). The columns represent individual tissue samples covering 3 breast cancer subtypes: Luminal A (*red*), Luminal B (*yellow*), and Triple negative (*purple*) as well as normal samples (*blue*). The rows are labeled with individual gene symbols

Next, the gene expression fold changes were further constrained to be ≥ 10 while still keeping the corrected *p*-value < 0.05. The distributions of the fold changes and *p*-values of genes in each subgroup were shown in Fig. 2 as volcano plots. Moreover, 28 genes were identified to show fold changes ≥ 10 in all three breast cancer subtypes. Figure 3 shows the heat map [31] representing the gene expression profiling of these 28 genes. Cancer samples are shown on the left grouped by breast cancer subtypes, while normal controls are displayed on the right. The detailed fold change values of these 28 genes are listed in Table 2. The gene regulation patterns of all 28 genes were consistent among the three breast cancer subtypes. Interestingly, most of these genes (25 genes) were down-regulated, and only 3 genes (COL10A1, MMP11, and TUBB3) were up-regulated in cancer tissues.

**Table 2:** List of 28 genes involved in breast cancer ^a^

	Gene		Fold change		
	Symbol	Description	Luminal A	Luminal B	Triple negative
1	GRIA4	Glutamate receptor, ionotropic, AMPA 4	−36.73	−10.59	−22.19
2	PIGR	Polymeric immunoglobulin receptor	−89.25	−24.54	−55.02
3	TUBB3	Tubulin, beta 3 class III	11.60	13.94	22.60
4	SLC26A3	Solute carrier family 26 (anion exchanger), member 3	−11.33	−10.86	−16.08
5	LINC00844	Long intergenic non-protein coding RNA 844	−15.33	−25.99	−13.74
6	FIGF	c-Fos induced growth factor	−49.22	−37.19	−18.18
7	SMYD1	SET and MYND domain containing 1	−31.85	−12.84	−17.43
8	CXCL2	Chemokine (C-X-C motif) ligand 2	−30.24	−13.34	−16.54
9	ALB	Albumin	−76.81	−54.76	−109.05
10	DRD2	Dopamine receptor D2	−19.79	−15.78	−10.30
11	DLK1	Delta-like 1 homolog	−82.62	−47.07	−19.17
12	RNF186	Ring finger protein 186	−64.51	−23.30	−61.59
13	C2orf40	Chromosome 2 open reading frame 40	−69.81	−13.55	−18.39
14	OXTR	Oxytocin receptor	−48.00	−24.36	−28.75
15	DAPL1	Death associated protein-like 1	−20.83	−20.53	−25.10
16	COL10A1	Collagen, type X, alpha 1	36.84	13.82	39.31
17	FGFBP1	Fibroblast growth factor binding protein 1	−50.79	−50.39	−17.39
18	NTRK2	Neurotrophic tyrosine kinase, receptor, type 2	−23.96	−15.75	−48.94
19	SCARA5	Scavenger receptor class A, member 5	−21.61	−19.18	−39.67
20	DLK2	Delta-like 2 homolog	−28.32	−11.49	−15.35
21	AFP	Alpha-fetoprotein	−29.03	−14.92	−42.89
22	HPSE2	Heparanase 2	−29.29	−20.28	−15.26
23	CAPN6	Calpain 6	−28.50	−38.50	−13.62
24	LINC00640	Long intergenic non-protein coding RNA 640	−18.13	−22.01	−12.84
25	LRRC3B	Leucine rich repeat containing 3B	−13.77	−21.02	−13.11
26	IL22RA2	Interleukin 22 receptor, alpha 2	−12.01	−22.72	−14.56
27	KCNJ16	Potassium inwardly-rectifying channel, subfamily J, member 16	−35.51	−11.58	−17.09
28	MMP11	Matrix metallopeptidase 11	24.75	12.45	50.25

^a^Genes with corrected *p*-value < 0.05 and fold changes ≥ 10 in all the 3 subtypes using GeneSpring 12.6 software

### Real-time RT-qPCR validation

Gene selection in real-time RT-qPCR validation is based on the selection criteria of corrected *p*-value < 0.05 and fold changes ≥ 10 and the relevance of genes to breast cancer progression. It is interesting to notice that both MMP11 and HPSE2 appear in the 28 top genes list in Table 2. As illustrated in Fig. 6, MMPs, HPSE, HPSE2 are closely involved in cancer cells’ invasion and metastasis. It has been previously documented that MMPs and HPSE play essential roles in breast cancer [32, 33]. However, the close relationship between MMP11 and HPSE2 has not been reported, which brings new insight into the breast cancer field. From our gene microarray data, MMP11 was found to be up-regulated while HPSE2 was down-regulated in breast cancer compared with normal control (Fig. 7). Therefore, MMP11 and HPSE2 were selected for real-time RT-qPCR validation to investigate their potential roles as a gene set in breast cancer progression.

**Fig. 4:**
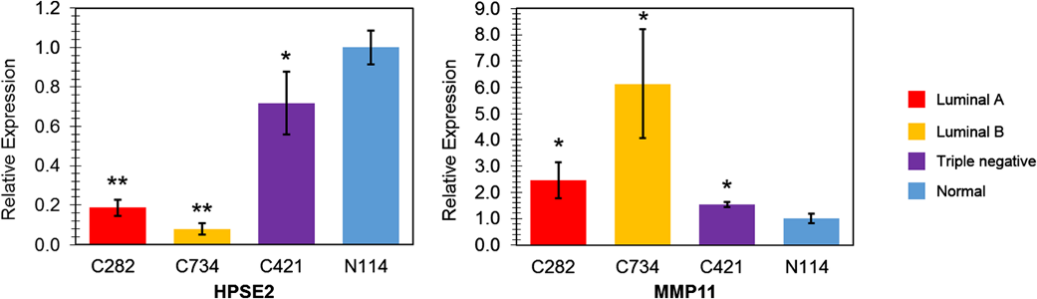
Validation of expression of HPSE2 and MMP11 using RT-qPCR. Four samples (C282, C421, C734, and N114) were picked from the training set, representing Luminal A, Luminal B, Triple negative, and normal tissue respectively. Fold changes of gene expression were calculated with the 2^-ΔΔC^_T_ method, using *β*-actin as the house keeping gene. Results were shown as mean ± SEM from triplicates (*n* = 3). *p < 0.05 compared with N114, **p < 0.001 compared with N114

Four tissue samples were first picked from the training set, C282, C421, C734, and N114, representing Luminal A, Luminal B, Triple negative, and normal tissue respectively. As shown in Fig. 4, the expression of HPSE2 in all the three cancer tissues were down-regulated compared with the expression in normal tissue. On the other hand, the levels of MMP11 in all the 3 cancer tissues were elevated compared with that in normal tissue. The RT-qPCR results for these samples were consistent with our gene expression microarray data (Table 2).

**Fig. 5:**
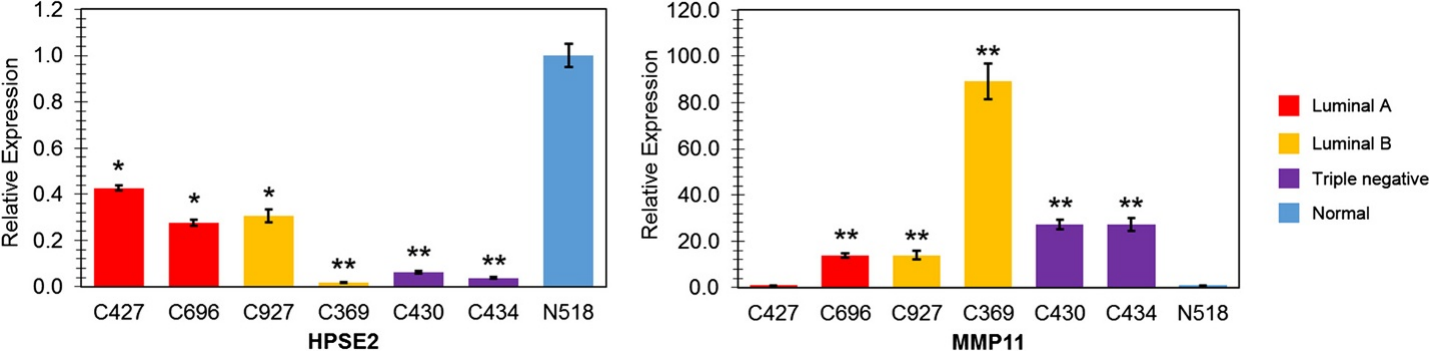
Validation of expression of HPSE2 and MMP11 using RT-qPCR. Seven samples were picked from the training set, representing Luminal A (C427 and C696), Luminal B (C927 and C369), Triple negative (C430 and C434), and normal tissue (N518) respectively. Fold changes of gene expression were calculated with the 2^-ΔΔC^_T_ method, using *β*-actin as the house keeping gene. Results were shown as mean ± SEM from triplicates (*n* = 3). *p < 0.05 compared with N518, **p < 0.001 compared with N518

To further validate the reliability of our gene array data, another 7 samples were randomly selected from the testing set (Fig. 1), including subtype Luminal A (C427 and C696), Luminal B (C927 and C369), Triple negative (C430 and C434), and normal control (N319). The results were shown in Fig. 5, which confirmed the gene expression profile for HPSE2 and MMP11 from microarray data. Validation with other samples from the testing set is still ongoing while our initial testing results demonstrated the reliability of the gene expression profiling generated by our genearray data.

## Discussion

### Gene expression microarray as a powerful tool to identify biomarkers in breast cancer

Unlike most traditional molecular biology tools, which only allow study of a single gene or a very small set of genes, gene expression microarrays provide a comprehensive overview of the entire transcriptional activity in a biological sample. As a result, gene expression microarrays significantly facilitate and accelerate the discovery of novel and unexpected functional roles of genes. This powerful tool has been applied to a broad range of applications, including discovering novel disease biomarkers and developing new diagnostic tools [20].

In the current study, 32 RNA samples from breast cancer patients as well as normal controls were employed as a training set. The expression profiling of as many as 50,739 genes in these samples were examined simultaneously using the newly developed Agilent Sure Print G3 Human Gene Expression Microarray technology, which provided comprehensive coverage of genes and transcripts with the most up-to-date genomic content. Distinct expression patterns between cancer and normal samples were identified (Fig. 3). Furthermore, there were 28 genes that have fold changes (the expression levels in cancer samples compared with normal controls) larger than 10 and *p*-value less than 0.05 (Table 2, Fig. 3), suggesting their important roles in cancer development and as biomarkers in breast cancer diagnostics. Moreover, the RT-qPCR results of several genes from both the training set and the testing set displayed good consistency with the results obtained from gene expression microarray, further indicating the accuracy and reliability of this technology (Fig. 4, 5).

### MMP11 and HPSE2 as a biomarker gene set in breast cancer

One of the main characteristics of breast cancer is its significantly higher capacity of invasion and metastasis. Most breast cancers are invasive, or infiltrating [1, 9], breaking through the ductal of glandular walls where they originated and growing into surrounding breast tissues. The invasive capacity is influenced by interactions between cancer cells and their extracellular matrix (ECM) components. During invasion and metastasis, tumor cells destruct the basement membrane (BM) and migrate into the connective tissue. The degradation of ECM and BM may further release and activate ECM-bound cytokines and ECM fragments that modulate cell growth, migration and angiogenesis [34].

**Fig. 6:**
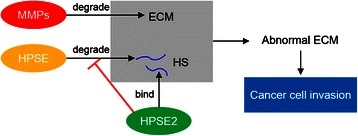
The involvement of MMPs, HPSE, and HPSE2 in ECM degradation and cancer cell invasion. MMPs are capable of degrading all kinds of ECM proteins. HPSE degrades heparan sulfate (HS), which is present in the ECM in the form of proteoglycans. HPSE2 lacks HS-degrading activity but remains high affinity towards HS. Abnormal ECM dynamics lead to deregulated cancer cell proliferation and invasion

Evidences now suggest that matrix metalloproteinase (MMP) and heparanase (HPSE) play important roles in degrading BM and ECM (Fig. 6). MMPs are zinc-dependent endopeptidases, which are capable of degrading all kinds of ECM proteins. The overexpression of many MMP family members, such as MMP1, MMP2, MMP7, MMP9, and MMP11 has been found to be involved in cancer progression [33, 35, 36]; therefore, the development of MMP inhibitors has become an effective strategy in clinical cancer therapies [37]. HPSE degrades heparan sulfate (HS), which is present at the cell surface and in the ECM in the form of proteoglycans. On the other hand, HPSE2, a homologue of HPSE, lacks HS-degrading activity. Nonetheless, HPSE2 remains capable of high-affinity interaction with HS. Therefore, HPSE2 acts as a competitive binder with HPSE for HS, thereby showing anti-metastatic features [32, 38]. The correlation between the expressions of MMP9 and HPSE in cancer progression; has been observed previously in different types of cancer [34, 39, 40]. However, the close relationship between MMP11 and HPSE2 was first discovered and further investigated in our study.

**Fig. 7:**
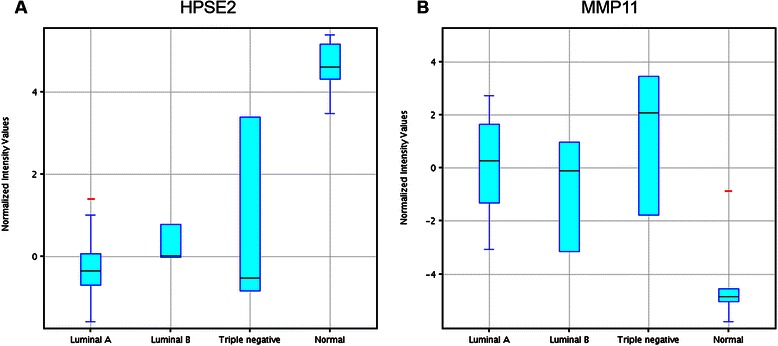
Box-and-Whisker plots. The gene expression levels of HPSE2 (**a**) and MMP11 (**b**) from the 32 samples in the training set covering Luminal A (*n* = 19), Luminal B (*n* = 3), Triple negative (*n* = 3) and normal control (*n* = 7) were shown in the box-and-whisker plots. The plots were generated using GeneSpring 12.6 software. The correlation of fold changes (FC) and normalized intensity (NI) values were calculated using the formula FC (X_n_) = 2 ^ [averaged NI (X_n_)-averaged NI (X_Control_)]. X: individual genes; n: breast cancer subtypes; NI (X_n_): Normalized intensity of gene X in subtype n; NI (Control): normalized intensity of gene X in normal samples

**Fig. 8:**
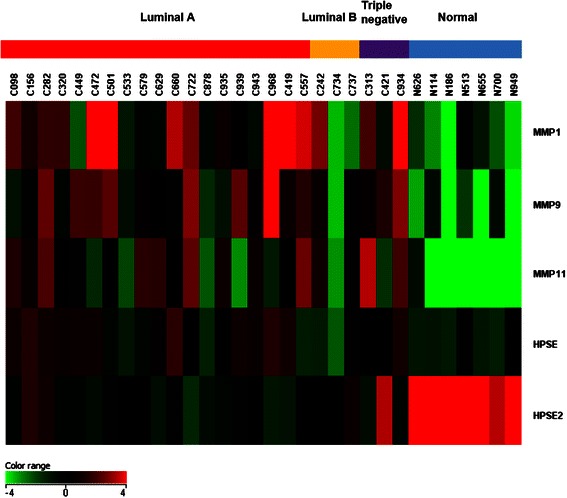
Heat map showing gene expression patterns of MMP1, MMP9, MMP11, HPSE, and HPSE2. The heat map indicates up-regulation (*red*), down-regulation (*green*), and mean gene expression (*black*). The columns represent individual tissue samples covering three breast cancer subtypes: Luminal A (*red*), Luminal B (*yellow*), and Triple negative (*purple*) as well as normal samples (*blue*). The rows are labeled with individual gene symbols

As shown in Table 2, both MMP11 and HPSE2 were found on the top 28 genes list with fold changes ≥ 10. Consistent with the above notion, our gene expression profiling results showed that while MMP11 was up-regulated by 12.45 to 50.45 folds in breast cancer tissue samples compared with normal controls, HPSE2 was down-regulated by −15.26 to −29.29 folds (Table 2). The Box-and-Whisker plots for the normalized intensity (NI) values of these 2 genes are shown in Fig. 7, which highlights the important features and shows the variations of the gene expression in each subgroup. In addition, another 2 well-studied genes in the MMPs family, MMP1 and MMP9, were also found to be up-regulated (2.22–21.18 and 3.56–21.41 folds, respectively) from our gene expression microarray data, although they were not in the top 28 genes list. With regards to HPSE, it was slightly up-regulated in Luminal A and Triple negative samples (2.40 and 2.48 folds, respectively), but down-regulated (−1.33 folds) in Luminal B subtype, suggesting that HPSE was not a suitable biomarker. The heat map for these genes is shown in Fig. 8.

Our analysis further identified a negative correlation between the expression of MMP11 and HPSE2 with a correlation coefficient of −0.72 (p < 0.0001, calculated by GraphPad Prism 6). More importantly, the gene regulation of MMP11 and HPSE2 was validated using real-time RT-qPCR with RNA samples from both training and testing sets (Figs. 3 and 4). All the results above suggest that MMP11 and HPSE2 can be used as a promising biomarker gene set in breast cancer. Given the synergetic effects of MMP11 and HPSE2, our findings may shed light on target-based anticancer drug design and development.

## Conclusion

Breast cancer is one of the most common cancers and the leading health crises for women today. Identification of mechanisms and biomarkers in breast cancer remains an urgent challenge [2, 41–45]. By applying state-of-the-art Agilent SurePrint G3 Human Gene Expression Microarray technology to clinical human tissue samples, we were able to obtain a comprehensive snapshot of the gene expression profile of breast cancer, providing informative data to identify novel biomarkers. Expressions of MMP11 and HPSE2, 2 genes closely involved in ECM-mediated cancer cell migration and angiogenesis, were found to be significantly different in breast cancer samples compared with normal controls. This important finding was further confirmed by real-time RT-qPCR. To the best of our knowledge, this is the first time that these 2 genes are demonstrated to act as a gene set in breast cancer. Our findings identify the negative correlation of MMP11 and HPSE2 in breast cancer progression, which provides novel insight into the optimization of breast cancer treatment. Based on our results, effective and targeted therapy for patients with different breast cancer subtypes can be designed and optimized for clinical application to more precisely identify and attack cancer cells by selectively inhibiting the expression of MMP11 or inducing the expression of HPSE2. The efforts to target those 2 genes in anti-breast cancer research with chemically synthesized molecules have already been initiated in our group [46].

### Availability of supporting data

This gene expression microarray data is deposited to the GEO repository (accession number: GSE57297) and this material is available free of charge via the Internet at http://www.ncbi.nlm.nih.gov/geo/query/acc.cgi?acc=GSE57297.

## Abbreviations

TNBC: Triple negative breast cancer
RT-q PCR: Reverse transcription quantitative polymerase chain reaction
ECM: Extracellular matrix
MMP: Matrix metalloproteinase
HPSE2: Heparanase 2
HS: Heparan sulfate
